# Surgical management of pes planus in children with cerebral palsy: A systematic review

**DOI:** 10.1177/18632521221112496

**Published:** 2022-09-02

**Authors:** Poppy MacInnes, Thomas L Lewis, Cora Griffin, Michela Martinuzzi, Karen L Shepherd, Michail Kokkinakis

**Affiliations:** 1 GKT School of Medical Education, King’s College London, London, UK; 2 Evelina Children’s Hospital, St Thomas’ Hospital, London, UK

**Keywords:** Flatfoot, pes planovalgus, pes planus, surgery, cerebral palsy, pediatrics, orthopedics

## Abstract

**Purpose:**

Pes planus (or flatfoot) is the most common deformity in children with cerebral palsy. There are several surgical interventions used to treat it: single calcaneal osteotomies, extra-articular arthrodesis, double calcaneal osteotomy, calcaneo-cuboid-cuneiform osteotomy, intra-articular arthrodesis, and arthroereisis. There is currently no evidence on optimal treatment for flatfoot in children with cerebral palsy. Our purpose is to systematically review studies reporting complications, recurrence rates, and radiological outcomes of the surgical management of flatfoot in children with cerebral palsy.

**Methods:**

Five databases were searched to identify studies published from inception until July 2021, with keywords relating to flatfoot, cerebral palsy, and surgical interventions. We included prospective, retrospective, and comparative study designs in the English language. Data was extracted and tabulated in duplicate into Excel, and analysis was conducted using Python SciPy.

**Results:**

In total, 1220 studies were identified of which 44 met the inclusion criteria, comprising 2234 feet in 1364 patients with a mean age of 10.3 years and mean follow-up of 55.9 months. Radiographic outcomes showed improvement with all procedures; complications and recurrence rates were too poorly reported to compare. Only 6 (14%) studies were assessed as a low risk of bias. There was substantial heterogeneity of outcome measures.

**Conclusion:**

There is a lack of high-quality, comparative studies assessing the radiological outcomes, complications, and recurrence rates of surgical alternatives to treat flatfoot in children with cerebral palsy. There is currently no clear evidence on optimal surgical treatment.

**Level of evidence:**

IIa based on Oxford Centre for Evidence-based Medicine.

## Introduction

Pes planus (also known as flatfoot or pes planovalgus) is the most common foot deformity in children with cerebral palsy (CP).^1^ The pathology develops due to the lateral displacement of the navicular, causing loss of the medial longitudinal arch, talar head uncovering, and talar prominence in the medial foot.^1^ The condition can be categorized into flexible and stiff.^2^ Flexible deformity involves preservation of the arch when sitting, extending the great toe or standing on tiptoes; stiff deformity involves a flat arch with limitation of motion during weight-bearing and non-weight-bearing, and is more difficult to treat.^3^ Higher functioning, ambulatory patients (Gross Motor Function Classification System (GMFCS) I–III) usually present with flexible flatfoot, whereas stiff flatfoot is more common in adolescents with lower functional ability (GMFCS IV–V).^4^ The deformity usually worsens during late childhood and can cause significant pain, pressure ulcers, and difficulty walking or wearing shoes.^3,5^ Surgical management is indicated when conservative measures have failed.

There are several surgical interventions used to treat pes planus but no guidelines on how to choose between them. Extra-articular arthrodesis (EAA) or single calcaneal osteotomies (SCO) are commonly used to treat children with milder, flexible deformities, and lower GMFCS levels. SCO includes calcaneal lateral column lengthening (LCL) and calcaneal slide (CS) with concomitant soft tissue procedures (peroneus brevis lengthening, tibialis posterior shortening, and talonavicular joint capsule reefing), and occasionally a medial cuneiform osteotomy. Double calcaneal osteotomy (DCO) and calcaneo-cuboid-cuneiform “triple C” osteotomies (TCO) have been used to treat moderate-to-severe deformities that would likely recur with SCO and EAA.^6^ Intra-articular arthrodesis (IAA) is an invasive procedure that has been reserved for children with GMFCS IV or V and/or severe, stiff deformities.^1,5^ Subtalar arthroereisis (SA) is a non-fusion procedure that has recently received renewed interest in the literature as an alternative to SCO and EAA.^2^

The purpose of this study is to systematically review the literature regarding the radiological outcomes, complications, and recurrence rates of current surgical management of flatfoot in children with CP.

## Methods

This systematic review was reported according to Preferred Reporting Items for Systematic Reviews and Meta-Analysis (PRISMA 2020) checklist and the AMSTAR 2 critical appraisal tool.^7,8^ The protocol was prospectively registered on PROSPERO CRD420201239285.^9^ The authors declare no conflict of interest relevant to this work.

### Search strategy

A literature search was conducted using the online Cochrane Library, EMBASE, MEDLINE, Web of Science, and PubMed databases, using the following terms: ((cerebral palsy)) AND (((pes planus) OR (flat foot) OR (pes planovalgus)) OR ((calcaneal) OR (calcaneus) OR (calcaneum) OR (slide) OR (double) OR (heel) AND (osteotomy) OR ((fusion) OR (arthrodesis) OR ((arthroereisis) OR ((Grice Green) OR (Grice-Green) OR ((lateral column lengthening) OR (MOSCA))). No limitations were placed on gender, date, or language. All results from inception until July 31, 2021 were included (Appendix 1).

### Inclusion criteria

We included all prospective, retrospective, and comparative study designs (randomized controlled trials (RCTs), case studies, cohort studies, and case-controlled studies) reporting original/primary data on one or more of the outcomes of interest. A scoping review identified a significant lack of RCTs on this subject, thus including non-randomized studies was necessary for an all-encompassing review.

### Exclusion criteria

We excluded duplicate articles, cost-effectiveness studies, and studies not reporting on primary data (such as review articles, editorials, discussions, commentaries, letters, and conference abstracts). We excluded studies not reporting data on radiographic outcomes, complications, and recurrence rates. Studies where data for pediatric patients with CP was not readily separable from other participants and where surgery was not the primary intervention were excluded on the grounds of not being relevant to the aims of the review.

### Participants

Children with CP and symptomatic pes planus were included. Studies with a mean age of participants below 18 years of age were included. Children without CP treated for foot deformities other than pes planus were not included.

### Intervention

The intervention was operative surgical management to treat symptomatic pes planus where conservative management had failed. The specific procedures identified by a scoping review included calcaneal LCL, EAA, CS, DCO, calcaneo-cuboid-cuneiform TCO, IAA, and SA. Data on variations of these procedures and any soft tissue procedures performed in conjunction was also extracted.

LCL is a procedure originally described by Evans that equalizes both columns in the foot via an osteotomy of the calcaneus bone approximately 1.5 cm proximal to the calcaneocuboid joint; as the lateral column is shorter in flatfoot, this equalization corrects forefoot abduction and restores the medial longitudinal arch.^10^ Mosca popularized the procedure by adding the soft tissue procedures of peroneus brevis lengthening, tibialis posterior shortening, and talonavicular joint reefing, and a plantar closing-wedge osteotomy of the medial cuneiform.^11^

EAA, originally used by Green and first reported by Grice in 1952, involves the extra-articular positioning of a structural autograft (either fibula or anterior tibia) between the talus and the calcaneus.^12^

CS is the medial displacement of the posterior part of the calcaneus, thus creating a compensating deformity to improve the heel valgus and normal weight-bearing.^13^ DCO is a combination of LCL and CS.

TCO is a versatile procedure that allows correction at the fore-, mid- and hindfoot by three osteotomies: a CS, an opening-wedge cuboid osteotomy, and a plantar flexion closing-wedge osteotomy of the medial cuneiform.^14^

SA involves the insertion of an implant into the sinus tarsi or adjacent to it to prevent talonavicular impingement which consequently blocks and corrects excessive eversion movements of talus and calcaneus, and maintains the subtalar joint in a more neutral position.^2^

Finally, IAA is a fusion of one or all of the joints of the hind- or midfoot, usually undertaken as a triple arthrodesis involving the talonavicular, subtalar, and calcaneocuboid joints.^15^

### Comparators

There is currently no gold standard for the surgical management of flatfoot in children with CP. We included papers that surgically managed flatfoot by LCL, CS, DCO, TCO, EAA, IAA, and SA using traditional or modified techniques. Non-surgical management of flatfoot was excluded.

### Outcomes

Primary outcomes were radiographic angles, complications, and recurrence rates. The radiographic angles included were most commonly used to assess flatfoot: anterior–posterior talocalcaneal (AP TC), anterior–posterior talo-first metatarsal (AP T1MT), and talonavicular coverage (TNC) angles; and lateral talocalcaneal (Lat. TC), lateral talo-first metatarsal (Lat. T1MT), calcaneal-first metatarsal (C1MT), and calcaneal pitch (CP).^2^ Gait analysis and clinical outcomes were not assessed, as gait analysis is infrequently reported in studies and there is no current standardized tool for assessing clinical outcomes for each surgical procedure.

### Data extraction

Study selection was performed in duplicate (P.M., C.G., and P.M., M.M.), and data extraction was performed in duplicate (P.M., C.G., and P.M., M.M.). Discrepancies over the inclusion of any study or data extraction were resolved by consensus or arbitration by senior authors (T.L.L. and M.K.).

For every article, the following data was extracted based on a scoping literature review:

Article demographic details (number of authors, title, year published, level of evidence (1–5), funding sources). Patient demographic details (number of patients, number of feet operated on, gender of patients, mean age, and age range of patients; GMFCS level of disability; mean follow-up (months/years) and range of follow-up).

Surgery details: type of surgery, indication for surgery, and concurrent procedures.

Radiographic outcomes: AP TC, AP T1MT, and TNC angles; and Lat. TC, Lat. T1MT, C1MT, and calcaneal pitch.

Complications and recurrence rates

Gait analysis and pedobarographic outcomes were not tabulated or synthesized due to the heterogeneity of the reporting between the studies.

### Assessment of methodological quality

The level of evidence and methodological quality of included studies was assessed using the MINORS criteria.^16^ A MINORS score of 16/16 or 24/24 was deemed high quality (and low risk of bias), 10–15/16 or 15–23/24 was deemed moderate quality (and moderate risk of bias), and a score of < 10/16 or < 15 was deemed low quality (and at high risk of bias) based on previous studies that used these scores. The articles were independently assessed by three authors (P.M., C.G., and M.M.) with a senior author settling any disagreement (T.L.L.). P.M. recorded sources of funding for individual studies included in the review.

### Statistical analysis

Where data was provided, weighted means of radiographic outcomes and recurrence rates of the surgical procedures were calculated. An independent *t*-test was used to compare the weighted means. All data analysis was conducted using Python SciPy.^17^ Radiographic results were considered statistically significant when reported to have a *p*-value of less than 0.05.

## Results

### Literature search

**Figure 1.**
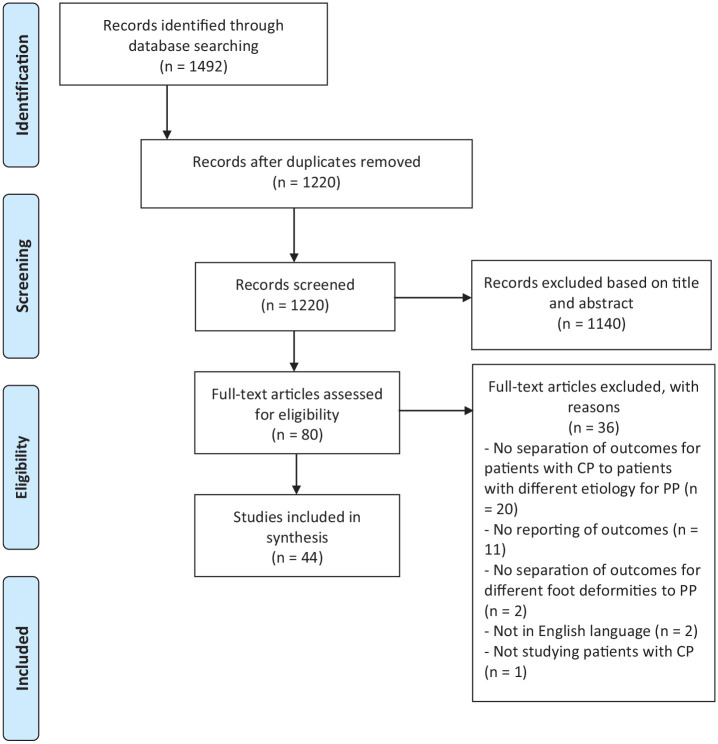
A prisma flow diagram for the systematic review detailing the database searches, the number of abstracts screened and the full texts reviewed. CP = cerebral palsy; PP = pes planus.

The initial search yielded 1220 articles for review after duplicates were removed as shown in Figure 1. Review of titles and abstracts identified 80 articles for full-text screening, of which 44 met the inclusion criteria. The main reasons for excluding articles at this stage were “no reporting of outcomes” (*n* = 11, 31%) and “no separation of outcomes for patients with CP to patients with different etiology for pes planus (PP)” (*n* = 20, 56%).

### Study and patient characteristics

The search identified 10 comparative studies (23%): 8 of these were retrospective comparative studies (18%) and 1 was a prospective, randomized design (2%). Of the remaining studies, 7 were prospective case series (16%) and 27 were retrospective case series (63%). The study characteristics and outcomes of the papers included can be seen in Table 1 and summarized in Table 2.

**Table 1.** Table of included studies.

Study	Study design (Oxford level of evidence)	Procedure type	No. of patients (M: F)	No. of feet	Mean age in years (range)	GMFCS or level of disability	Outcomes used	Mean follow-up period in months (range)	MINORS score (quality)
Aboelenein et al.^18^	Prospective case series (4)	Calcaneal LCL	15 (5:10)	22	11.5 (8.3–14.5)	II–III	AP T1MT, AP TC, Lat T1MT, Lat TC, CP, and Dogan’s scale	31 (26–44)	12 (good)
Abu-Faraj et al.^19^	Retrospective case series (4)	EAA	12 (8:4)	17	13.1 ± 2.6	Ambulatory	3D gait analysis and plantar pressure measurements	12	8 (poor)
Adams et al.^20^	Retrospective comparative study (3)	Calcaneal LCL	42 (19:23)	61	9 (6.3–13.9)	Not stated	Lat. TC, AP and Lat. T1MT, Lat. TNC, CP, Lawrence and Kellgren criteria for OA.	70 (41–102)	16 (poor)
Ahmed et al.^21^	Prospective, randomized, comparative study (2)	Calcaneal LCL, SA	35	57	9	I–III	AP TNC; AP and Lat T1MT, AP and Lat. TC, CP; talar declination angle Yoo et al. clinical score, patient satisfaction, orthosis, shoes	15.6 (12–22)	18 (poor)
Alman et al.^22^	Retrospective case series (4)	EAA	29	53		Ambulatory	AP and Lat. TC, AP TNC Clinical and radiographic assessment of ankle and subtalar alignment, braces, skin calluses	106.8	10 (poor)
Aly et al.^23^	Prospective case series (4)	DCO	16 (9:7)	24	10.74 (6–16)	I–III	AP and Lat. CP, TC, T1MT Talar head uncoverage (%) Clinical heel valgus	33.5 (24–48)	10 (poor)
Andreacchio et al.^24^	Retrospective case series (4)	Calcaneal LCL	15	23	9.1 (6.2–17.8)	Ambulatory	Lat. T1MT, Lat., AP TNC Cosmesis, walking distance, walking support, pain	49.2 (27.6–61.2)	10 (poor)
Bhan and Malhotra^25^	Retrospective case series (4)	EAA	10	16	6.2 (4–11)	Not stated	Lat. TC, heel valgus alignment Mobility, pain	43.2 (24–72)	10 (poor)
Barrasso et al.^26^	Retrospective case series (4)	EAA	26 (17:9)	40	10.5 (3.5–14.9)	Ambulatory and non-ambulatory	Lat. TC Clinical evaluation of ambulatory status, physical examination	30 (16–53)	10 (poor)
Bourelle et al.^27^	Retrospective case series (4)	EAA	17 (9:8)	26	5.4 (3.8–8.6)	Ambulatory	AP and Lat. TC, Lat. talar declination, Heel valgus alignment Pain, walking assessment, type of shoe, footprint analysis, callus formation, physical examination	243 (207–276.6)	10 (poor)
Cho et al.^28^	Retrospective case series (4)	Calcaneal LCL	44 (27:17)	77	10.5	I–IV	Lat TC, AP and Lat. T1MT, CP	61.2	12 (good)
Costici et al.^29^	Retrospective case series (4)	IAA	103 (64:39)	175	14.7 (12–20)	I–IV	AP TC, AP TNC, Costa Bertani angle Visual analogue scale for pain, GMFCS scale.	62.4 (12–112)	10 (poor)
de Moraes Barros Fucs et al.^30^	Retrospective case series (4)	IAA	21 (13:8)	35	16 (8–29)	II–IV	Inclination of the calcaneus angle, Lat. TC Pain, walking assessment, need of braces, type of shoe, physical examination	58 (30–90)	7 (poor)
Elbarbary et al.^31^	Prospective case series (4)	SA	23 (16:7)	46	8.6 (6–12)	I–III	Lat-TCA, heel valgus alignment, OxFAQ-C (physical, school and play, emotional, shape of foot, shoe wear, walking ability)	36.7 (24–40)	12 (good)
El-Hilaly et al.^32^	Prospective case series (4)	TCO	12 (7:5)	18	9.7 (5.1–15.3)	I–IV	Lat. TC, Lat. T1MT, TNC, CP, dorsoplantar T1MT Pedobarography	4.25 (2.5–6.5)	11 (poor)
Engström et al.^33^	Prospective case series (4)	EAA	16	27	6 (3–12)	Not stated	Subtalar stability, corrected valgus hindfoot, gait improvement, radiographic analysis of union	39.6 (12–96)	7 (poor)
Ettl et al.^34^	Retrospective case series (4)	Calcaneal LCL	19 (12:7)	28	8.6 (4–18)	Ambulatory and non-ambulatory	Lat. T1MT, Lat. Horizontal angle, CP Mosca’s clinical criteria AOFAS ankle-hindfoot scale	51.6 (12–103.2)	10 (poor)
Güven et al.^35^	Retrospective case series (4)	EAA	11 (5:6)	15	10.7 (6–15)	I–IV	AP and Lat. TC, AP and Lat. T1MT, CP Walking, pain, skin calluses, orthoses, shoes, and survey	24 (9–39)	9 (poor)
Huang et al.^36^	Retrospective comparative study (3)	Calcaneal LCL ± medial column stabilization via talonavicular arthrodesis	21 (8:13)	37	11 (4.9–16)	II–III	AP. TNC Mosca’s radiographic and clinical criteria, Yoo et al. criteria	29.4 (13–63.6)	17 (poor)
Jeray et al.^37^	Retrospective case series (4)	EAA	28 (18:10)	52	7.4 (5–12)	Not stated	Lat. TC Survey	41 (27–78)	10 (poor)
Kadhim et al.^38^	Retrospective comparative series (3)	IAA, Calcaneal LCL	78 (43:35)	138	11.9 (4.7–18.3)	I–IV	Lat. TC, Lat. T1MT, CP Gait and kinematic analysis; pedobarography	60 (12–184.8)	15 (poor)
Kadhim et al.^39^	Retrospective comparative study (3)	IAA, Calcaneal LCL	24	43	11 (4.7–18.3)	I–IV	Lat TC, Lat. T1MT, CP Gait and kinematic analysis, pedobarography	130.8 (75.6–184.8)	15 (poor)
Kubo et al.^40^	Retrospective case series (4)	SA	11	19	9.2 (5–13)	II–III	AP and Lat. TC, AP and Lat. T1MT, CP, lateral relative overlap of Os navicular and Os cuboideum	35.2 (7–100)	10 (poor)
Leidinger et al.^41^	Retrospective case series (4)	EAA	35 (20:15)	51	7.8 (3.9–14.4)	Ambulatory	Lat. TC, CP Heel valgus alignment, patient satisfaction, GMFCS level	271.2 (192–387.6)	10 (poor)
Luo et al.^42^	Retrospective case series (4)	Calcaneal LCL	20 (14:6)	30	11.9	II–IV	AP and Lat. T1MT, CP, AP and Lat. TC, AP TNC, Lat. talo-horizontal angle Foot pain, callosity, tolerance to a foot orthosis	30 (12–72)	10 (poor)
Mazis et al.^43^	Retrospective case series (4)	EAA	11 (7:4)	16	9.7 (6.4–12.3)	Not stated	AP and Lat. TC, AP and Lat. T1MT, TNC, CP, naviculocuboid overlap	43.2 (24–99.6)	9 (poor)
Molayem et al.^44^	Retrospective case series (4)	SA	15 (7:8)	27	12.1 (9.3–14.5)	Ambulatory	AP and Lat. TC Pain, loss of function	61.2 (26.4–111.6)	10 (poor)
Muir et al.^45^	Retrospective case series (4)	IAA	5 (3:2)	9	14 (11–17)	IV–V	Radiographic outcomes not specified Mobility, shoes, and braces	60 (52–69)	8 (poor)
Nahm et al.^46^	Retrospective comparative study (3)	Calcaneal LCL and either medial cuneiform dorsal opening-wedge osteotomy or medial cuneiform plantar flexion closing-wedge osteotomy	24 (14:10)	42	9.7 ± 3.4	I–III	CP, AP and Lat. T1MT, multi-segment foot modeling (MSFM) gait analysis, physical examination	14.4 (9.6–42)	16 (poor)
Narang et al.^47^	Prospective case series (4)	Calcaneal LCL	10	17	8–18 (11.13)	I–II	AP TC, AP TN, Lat. CP, C5MT, Lat. T1MT Heel valgus alignment and heel rise tests, video gait analysis	12	12 (good)
Noritake et al.^48^	Retrospective case series (4)	Calcaneal LCL	16 (10:6)	27	10.8 (5.8–14.5)	Ambulatory	AP TN, AP and Lat-T1MT, Lat. CP, Lat talo-horizontal angle Mosca’s clinical criteria	38.4 (24–60)	9 (poor)
Park et al.^49^	Retrospective comparative study (3)	EAA, Calcaneal LCL	47 (27:20)	81	8.1 (5.5–16.7)	II	AP and Lat. T1MT, AP and Lat. TC. CP Pedobarography	39 (26–61)	16 (poor)
Rethlefsen et al.^50^	Retrospective comparative study (3)	CS, Calcaneal LCL	72 (41:31)	119	11.1	I–III	Gait kinematics and kinetics Modified Yoo system for change in standing foot position Modified Clavien–Dindo system for complications	38.4	14 (poor)
Rhodes et al.^51^	Retrospective comparative study (3)	Calcaneal LCL	36	63	9.3 (4–18)	I–V	Lat. TC, AP and Lat. T1MT, AP TNC, CP Worth et al. radiographic xenograft incorporation grade	37.25 (21.2–53.7)	16 (poor)
Senaran et al.^52^	Retrospective case series (4)	IAA	138 (73:65)	253	12.7 (5–20)	I–V	Radiographic reporting on fusion and hardware Mobility, shoes, heel valgus alignment, skin calluses, pain	57.6 (24–132)	9 (poor)
Shore et al.^53^	Retrospective case series (4)	EAA	46 (28:18)	92	12.9 (7.8–18.4)	II–IV	Lat. TC, Lat T1MT, navicular cuboid overlap, Mobility scale	55 (30–90)	10 (poor)
Sung et al.^54^	Retrospective case series (4)	Calcaneal LCL	75 (51:24)	75	11 (5–30)	Not stated	AP T1MT, CP, TC, Lat. T1MT	37.2 (12–101)	12 (good)
Turriago et al.^55^	Retrospective case series (4)	IAA	32 (16:16)	59	13.9 (9–20)	Ambulatory	Lat and AP TC, Lat and AP T1MT, Gait analysis, satisfaction questionnaire	40 (18.3–66.7)	7 (poor)
Vlachou et al.^56^	Retrospective case series (4)	EAA	5 (2:3)	6	10.6 (9–14)	Ambulatory	Lat. TC, TNC physical examination; symptomatic feet	96 (24–180)	9 (poor)
Vlachou and Dimitriadis^57^	Retrospective case series (4)	EAA	9 (3:6)	12	11.7 (9–14)	Ambulatory	Lat. TC, Lat. TNC, evidence of fusion Appearance of the feet, heel valgus alignment, local symptoms	93.6 (48–180)	9 (poor)
Wen et al.^58^	Retrospective comparative study (3)	EAA, SA	26 (17:9)	44	8.5 (5–15)	I–II	AP TC, Lat. T1MT AOFAS-AH	30.1 (20–60)	16 (poor)
Yoo et al.^59^	Retrospective case series (4)	Calcaneal LCL	56	92	9.2 (4–17.2)	Ambulatory	Lat. TC, Lat. T1MT, Lat. CP Gait analysis, heel valgus alignment	62.4 (24–93.6)	9 (poor)
Yoon et al.^60^	Retrospective case series (4)	EAA	30 (21:9)	50	9 (5–18)	Ambulatory	AP and Lat. T1MT, AP and Lat. TCA, CP, Lat. C1MT Kinematic analysis	37 (26–49)	9 (poor)
Zeifang et al.^61^	Prospective case series (4)	Calcaneal LCL	32 (22:10)	46	11 (4–22)	Ambulatory	Lat. TC, Lat. T1MT, Lat. CP, AP T1MT, Costa Bertani angle Modified Phillips clinical score	66 (36–108)	12 (good)

AP TC: anterior–posterior talocalcaneal; AP T1MT: anterior–posterior talo-first metatarsal; Lat. T1MT: lateral talo-first metatarsal; Lat. TC: lateral talocalcaneal; CP: cerebral palsy; TNC: talonavicular coverage; MSFM: multi-segment foot modeling; C1MT: calcaneal-first metatarsal; GMFCS: Gross Motor Function Classification System; OA: osteoarthritis; AOFAS: American Orthopaedic Foot and Ankle Society; AOFAS-AH: American Orthopaedic Foot and Ankle Society Ankle-Hindfoot scoring system; OxFAQ-C: Oxford Ankle Foot Questionnaire for Children.

**Table 2.** Summart of included studies.

	Calcaneal LCL	CS	EAA	DCO	TCO	IAA	SA
No. of studies	17	1	16	1	1	7	5
Sample size (no. of feet)	784	119	539	24	18	634	140
GMFCS (I–V)	I–V	I–III	I–IV	I–III	I–IV	I–V	I–III
No. of comparative studies	6	1	2	0	0	2	2

LCL: lateral column lengthening; CS: calcaneal slide; EAA: extra-articular arthrodesis; DCO: double calcaneal osteotomy; TCO: triple calcaneal osteotomy; IAA: intra-articular arthrodesis; SA: subtalar arthroereisis; GMFCS: Gross Motor Function Classification System.

The studies included 2234 feet in 1364 patients with a mean age of 10.3 years (ranging from 3 to 30 years) and a mean follow-up of 55.9 months (ranging from 4.3 to 217.2 months). Studies included patients with a GMFCS level of I–V, with both stiff and flexible flatfoot deformities. There was a significant focus on ambulatory patients with GMFCS level I–III and a flexible flatfoot deformity (*n* = 33, 75%).

### Outcomes

A majority of the papers (75%, *n* = 33) reported on pre- and post-operative radiographic deformity correction outcomes. All of these papers clearly stated that the radiographs were weight-bearing. Overall, the radiographic angles showed significant improvement within normal range with the exception of the Lat. T1MT angle in LCL and the AP TC angle in IAA (Table 3).

**Table 3.** Radiographic outcomes summarised using the weighted mean for each procedure.

	AP TC	Lat. TC	AP T1MT	Lat. T1MT	CP	AP TC	Lat. TC	AP T1MT	Lat. T1MT	CP
IAA	42.9	48.2	25.7	22.2	12	33.9	31.6	5.3	8.7	12.8
LCL	30	42.6	23.2	27.5	3.7	20.9	36.2	6	11	10.6
EAA	38.6	45.9	28.5	29.5	11	25.9	33.3	7.9	10	12
SA	34.4	47.2	26.5	26.5	5.2	27.5	31	5.11	5.5	9.8

AP TC: anterior–posterior talocalcaneal angle (normal range 15°–27°); Lat. TC: lateral talocalcaneal angle (normal range 25°–45°); AP T1MT: anterior–posterior talo-first metatarsal angle (normal range 3°–11°); Lat. T1MT: lateral talo-first metatarsal angle (normal range 2°–10°); CP: calcaneal pitch (13°–23°); IAA: intra-articular arthrodesis; LCL: lateral column lengthening; EAA: extra-articular arthrodesis; SA: subtalar arthroereisis.

The clinical outcomes were measured differently in all papers (Table 1). Similarly, of the 11 studies (25%) that reported on gait analysis, kinematics, and pedobarography, the heterogeneity of the measurements meant that a comparison of the data between studies was not possible.^19,32,38,39,46,47,49,50,55,59,60^

Given the heterogeneity in outcome measures between the studies and their general poor quality, it was not possible to synthesize a meta-analysis. A formal narrative synthesis of the results is provided following the Synthesis Without Meta-analysis (SWiM) reporting guidelines.^62^

### Complications and recurrence

Data regarding complication and recurrence rates was poorly reported (Table 4). There was no clear correlation between complication rates and GMFCS level or the severity of the deformity. Recurrence rates were highest in relation to LCL and CS, and lowest in relation to DCO, TCO, and SA (Table 5).

**Table 4.** Complications and recurrence rates.

Procedure	Study/modification to procedure	No. of feet	GMFCS/ ambulatory (A)/non-ambulatory (NA)	Complications (%)	Recurrence rate (%)
Calcaneal LCL	Aboelenein et al.^18^ Minor modification to Mosca, PBL, PLL, ATL	22	II–III	– Infection 4.5 – Under-correction 9	–
	Adams et al.^20^ – Group 1: Pin stabilization ATL, GR (PS) – Group 2: No stabilization ATL, GR (NS)	61	–	– Subluxation 86 (PS) – Subluxation 91 (NS) – Osteoarthritis 6 (NS)	–
	Ahmed et al.^21^ Evans, ATL, GR	29	I–III	– Pain 13.8 – Infection 6.9 – Under-correction 14	0
	Andreacchio et al.^24^ Mosca PBL, PLL, GR, ATL	23	A	– Non-union 13	25
	Cho et al.^28^ Minor modification to Mosca, PBL, ATL, GR	77	I–IV	– Subluxation 6.5 – Degenerative arthrosis 2.6	–
	Ettl et al.^34^ ATL, PBL TATT, PLL, open reduction of talonavicular joint	28	A and NA	– Infection 4	25
	Huang et al.^36^ – Group 1: CL, ATL, GR – Group 2: CL, medial column stabilization via talonavicular arthrodesis, ATL, GR	37 19 18	II–III	– Staple penetration into talonavicular joint 31.6 (Group 1); 11.1 (Group 2)	36.8 (Group 1); 16.7 (Group 2)
	Kadhim et al.^38^ GR, ATL, TATT	63	I–IV	–	–
	Kadhim et al.^39^ GR, ATL, TATT	15	I–IV	– Under-correction 20 – Hardware prominence requiring removal of hardware 47 – Pain 53	–
	Luo et al.^42^ Mosca, ATL, GR	30	II–IV	– Under-correction 43	0
	Nahm et al.^46^ Mosca, GR, ATL	24	I–III	–	–
	Narang et al.^47^ Mosca, PBL, PLR	17	I–II	– Paraesthesia sural nerve 5.9	5.88
	Noritake et al.^48^ Mosca, PBL	27	I–II	– Over-correction 4 – Under-correction 22 – Difficulty wearing brace 4	18.5
	Park et al.^49^ Mosca, GR, ATL PBL	37	II	–	–
	Rethlefsen et al.^50^ PBL	46	I–III	– Over-correction 13 – Transient neuropraxia due to concomitant hamstring lengthening < 23	64
	Rhodes et al.^51^ – Group 1: Bovine xenograft (X) GR, ATL – Group 2: Allograft (A) GR, ATL	63	I–V	– Pressure ulcer 24 (A) – Delayed union 2 (X) – Non-union 1 (X) – Revision surgery 2 (X)	15 (A) 13 (X)
	Sung et al.^54^ Minor modification of Evans, PBL, ATL GR	75	–	– Under-correction 28–40	–
	Yoo et al.^59^ Minor modification to Mosca, ATL, GR, PBL, PLR	92	A	– Subluxation 3.3 – Over-correction 7.6	4.3
	Zeifang et al.^61^ Evans, GR, PBL, RMC	46	A	– Hematoma 23 – Loss of correction 21 – Over-correction 9 – Subluxation 21 – Osteoarthritis 2	15.2
CS	Rethlefsen et al.^50^ RMC or TNF	73	I–III	– Over-correction 4 – Prolonged pain < 23 – Plantar hypersensitivity < 23	29
DCO	Aly et al.^23^ Mosca and medial slide; GR, PBL, TPA	24	I–III	– Under-correction 12.5 – Heel ulcer 6.25 – Chronic heel pain 6.25	0
TCO	El-Hilaly et al.^32^ PBL, GR, talonavicular reduction with capsular and tibialis posterior plication	18	I–IV	–	-
SA	Ahmed et al.^21^ ATL, GR	28	I–III	– Pain 25 – Under-correction 7	0
	Elbarbary et al.^31^ ATL, PBL, PLL, multilevel release	46	I–III	– Infection and removal of hardware 2.2	0
	Kubo et al.^40^ ATL, GR	19	II–III	0	0
	Molayem et al.^44^ – Group 1: Intra-sinus tarsi (IST) ATL – Group 2: Extra-sinus tarsi (EST) ATL	27	A	– Implant dislocation 21 (IST) – Implant fracture 15 (EST) – Implant dislocation 23 (EST)	0
	Wen et al.^58^ ATL	20	I–II	Pain 5	0
EAA	Abu-Faraj et al.^19^	17	A	–	–
	Alman et al.^22^ Modification of Grice	53	A	– Skin irritation 20.7 – Migration of smooth fixation wire 3.4 – Over-correction 13.7 – Revision surgery 3.4 – Tibial fracture at graft harvest site 3.4 – Ankle valgus 10.3	3.8
	Barrasso et al.^26^ Dennyson-Fulford	40	A and NA	– Heel ulcer 2.5 – Pseudoarthrosis (asymptomatic) 5	0
	Bhan and Malhotra^25^ Dennyson-Fulford, fibular dowel and screw ATL, PBT, Steindler’s plantar release	16	A	– Infection 6.3 – Hardware problems 31.2	0
	Bourelle et al.^27^ Chigot and Sananes modification of Grice ATL	26	A	– Infection 3.8 – Over-corrected 19.2 – Pain 26.6 – Graft resorption 27	0
	Engström et al.^33^ ATL	27	–	– Non-union 33 – Under-corrected 7	22
	Güven et al.^35^ Modification of Grice using subperiosteal fibular graft, GR	15	I–IV	0	0
	Jeray et al.^37^	52	–	– Non-union 12	3.8
	Leidinger et al.^41^ ATL PTL	51	A	– Graft slippage 1.96 – Revision surgery 1.96 – Under-correction 9.8 – Over-correction 7.84 – Shin bone fracture 3.92	3.92
	Mazis et al.^43^ Chigot and Sananes modification of Grice ATL	16	–	– Non-union 18.8 – Graft absorption 18.8	12.5
	Park et al.^49^ Modified Dennyson-Fulford, GR, ATL PBL	44	II	–	–
	Shore et al.^53^ Modified Dennyson-Fulford (dowel allograft)	92	II–IV	– Stable fibrous union 2.2	0
	Vlachou et al.^56^ Batchelor-Grice	6	A	0	0
	Vlachou and Dimitriadis^57^ Batchelor-Grice ATL	12	A	0	0
	Wen et al.^58^ Dennyson-Fulford, GR, ATL	22	I–II	– Pain 4.5 – Screw fracture 4.5	0
	Yoon et al.^60^ Modified Dennyson-Fulford ATL GR PBL	50	A	– Heel sore 6 – Necrosis of incision wound 4	0
IAA	Costici et al.^29^ Double arthrodesis Talonavicular + calcaneocuboid joint, GR	175	I–IV	– Infection 2.3 – Delayed union 3.4 – Hardware breakage 2.9 – Revision surgery 4.6 – Persistent pain 4	–
	de Moraes Barros Fucs et al.^30^	35	II–IV	– Non-union 50 – Pseudoarthrosis 37 – Pain 4.8 – Revision surgery 38.1	–
	Kadhim et al.^38^ Allograft and screw fixation, GR, ATL	75	I–IV	–	–
	Kadhim et al.^39^ Allograft and screw fixation, GR, ATL	28	I–IV	– Under-correction 29 – Hardware prominence requiring hardware removal 25 – Pain 11	–
	Muir et al.^45^ ATL	9	IV–V	– Non-union 11	–
	Senaran et al.^52^ ATL GR	253	I–V	– Infection 0.3 – Skin hypersensitivity 2.4 – Non-union 2 – Screw removal for irritation of tendons 2 – Pseudoarthrosis 0.8	2
	Turriago et al.^55^	59	A	– Pseudoarthrosis 12 – Under-correction 3.4 – Over-correction 1.7 – Revision surgery 12 – Pain 8.5	0

LCL: lateral column lengthening; DCO: double calcaneal osteotomy; TCO: triple calcaneal osteotomy; SA: subtalar arthroereisis; IST: intra-sinus tarsi; EST: extra-sinus tarsi; EAA: extra-articular arthrodesis; IAA: intra-articular arthrodesis ; PBL: peroneus brevis tendon lengthening; PLL: peroneus longus tendon lengthening; ATL: Achilles tendon lengthening; GR: gastrocnemius recession; PS: pin stabilisation; NS: nonstabilised; TATT: tibialis anterior tendon transfer; PLR: peroneus longus release; RMC: reefing medial capsule; NF: talonavicular joint fusion; TPA: tibialis posterior tendon advancement; PBT: peroneus brevis transfer; PTL: peroneal tendon lengthening.

**Table 5.** Weighted mean of recurrence rates for each procedure where data was provided.

LCL	CS	DCO	TCO	SA	EAA	IAA
18%	29%	0%	0%	0%	2.9%	1.6%

LCL: lateral column lengthening; CS: calcaneal slide; DCO: double calcaneal osteotomy; TCO: triple calcaneal osteotomy; SA: subtalar arthroereisis; EAA: extra-articular arthrodesis; IAA: intra-articular arthrodesis.

### Quality of studies included

**Figure 2.**
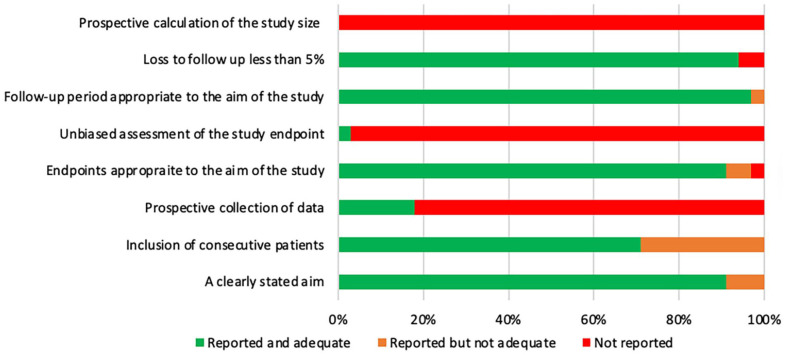
Bar chart demonstrating how non-comparative studies scored on MINORS.

**Figure 3.**
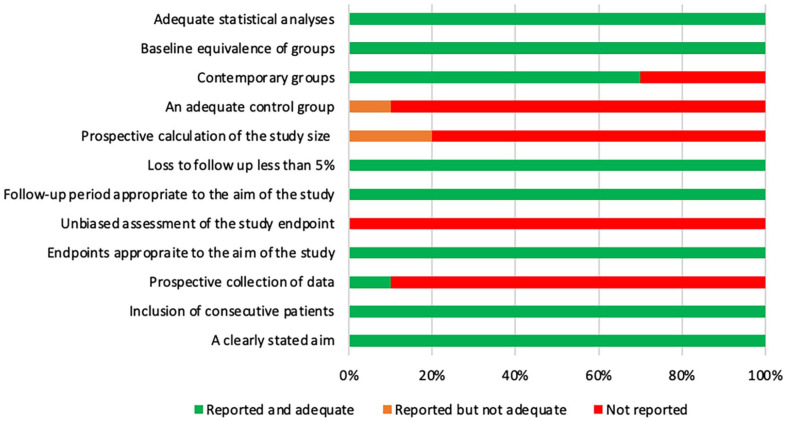
Bar chart demonstrating how comparative studies scored on MINORS.

The quality of the studies included was assessed according to the MINORS criteria (Figures 2 and 3). In total, 38 studies (86%) were assessed as having a high risk of bias, and 6 (14%) studies as having a low risk of bias.

## Discussion

This is the first systematic review of surgical management of pes planus in children with CP, covering 2234 operations from 44 papers. Overall, we found that substantial deformity correction was achieved by each surgical intervention. Based on the evidence, however, it is not possible to show that one intervention is superior to others.

There is a significant lack of studies on CS, DCO, TCO, IAA, and SA (Table 2). Most of the patients included in the studies in this review had flexible deformity with lower GMFCS levels; there is limited data to allow a proper assessment of treatment for moderate–severe flatfoot deformities. Ideally, studies would separate management of stiff flatfoot in GMFCS levels IV and V from flexible flatfoot in GMFCS levels I–III as it constitutes a different deformity. Many of the papers used levels I–IV or I–V, or described the patients as “ambulant” or “non-ambulant” making it difficult to undertake subgroup analysis as the data was not always clearly separated.

The radiographic outcomes show significant improvement is achievable by all surgical interventions. Severe deformity in patients with higher GMFCS levels is difficult to treat even with an invasive procedure such as IAA, and achieving long-term correction with LCL, EAA, CS, or SA is unlikely unless there is concomitant joint fusion.^34,36,50^ Four of the papers offered useful parameters for when a modified or more invasive procedure than LCL or EAA should be used to treat pes planus to avoid recurrence, but these papers were limited by the bias in the studies.^24,50,54,59^ Some studies combined techniques, such as Nahm et al.,^46^ which are valid surgical options and would merit further research.

Our study has highlighted the need for a standardized method of measuring clinical outcomes. Four of the studies on LCL used either Mosca or Yoo’s clinical criteria, the latter of which was adopted by Ahmed et al.,^21^ to assess the results of SA.^11,21,34,36,48^ These criteria could be combined in future and validated to compare different procedures, but could be adapted to incorporate activity levels to assess function. There was a notable lack of patient-reported outcomes in the studies which are essential to assess the effect of treatment on the patient’s quality of life. For example, relief of pain post-procedure is an important treatment outcome that could not be assessed in our review because it was either not measured at all or not in a consistent way. Standardized methods of measuring gait analysis, kinematics, and pedobarography are also needed given a general consensus in the included studies on the limited ability of radiographic outcomes to fully reflect the clinical picture.^19,32,38,39,46,47,49,50,55,59,60^

The poor reporting of complications could be improved by the use of clearer definitions, for example, avoiding the interchangeable use of terms such as “non-union” and “pseudoarthrosis,” or “under-correction” and “recurrence.” The high recurrence rates seen in LCL and CS procedures compared to other procedures reflect the high risk of bias in the studies rather than the actual difference in recurrence rates, and other procedures reported significant complications such as hardware complications for SA. Any conclusions on the comparison between treatments in regard to recurrence rates and complications would be misleading given the small size of the studies, short follow-up and reporting bias which may have hidden recurrence rates and complications.

The strengths of this review are that it includes papers on multiple interventions with a large sample size and a long follow-up. The 44 studies reported on a homogeneous population with minimal loss to follow-up. The main limitation of this review is the quality of the included studies which were mostly graded as “poor” and thus had a high risk of bias. The robustness of our synthesized results is difficult to assess given that data was often missing from the studies, especially regarding complications of the procedures. Furthermore, the heterogenous complication results meant that any analysis between the procedures is difficult to undertake. The retrospective case series did not have comparator interventions, meaning a potential lack of systematic pre- and post-operative assessment, and a high risk of bias in the clinical and radiographic outcomes. *P*-values were often not provided by papers to demonstrate whether radiographic outcomes were statistically significant, and often not combined with clinical outcomes to make them useful. The prospective and comparative studies were weakened by small study sizes and short follow-up periods. Longer follow-up periods are needed to reliably assess whether there are any degenerative changes to adjacent joints that can occur after fusion. Degenerative changes after IAA were not reported in the six studies with a mean follow-up of 71.4 months, thus a longer follow-up may be needed to exclude this outcome.^29,30,38,39,45,52,55^

## Conclusion

Pes planus is the most common foot condition for children with CP; a more robust evidence base is needed to provide guidance to surgeons on the optimal intervention for patients. Our review has highlighted the need for multi-center, large-scale, prospective, comparative studies, using standardized radiographic, clinical, and pedobarographic outcomes. Future studies should focus on interventions for patients with severe, stiff deformities, and higher GMFCS levels, and how the addition of fusion to procedures affects these patients in the long term.

## Appendix 1

**Table:**

Database: Ovid MEDLINE(R) ALL < 1946 to July 31, 2021> Search Strategy: 1 cerebral palsy.mp. [mp =ti, ab, hw, tn, ot, dm, mf, dv, kw, fx, dq, nm, kf, ox, px, rx, ui, sy] (70342) 2 (pes planus or flatfoot or pes planovalgus).mp. [mp =ti, ab, hw, tn, ot, dm, mf, dv, kw, fx, dq, nm, kf, ox, px, rx, ui, sy] (2997) 3 (lateral column lengthening or MOSCA).mp. [mp =ti, ab, hw, tn, ot, dm, mf, dv, kw, fx, dq, nm, kf, ox, px, rx, ui, sy] (4471) 4 ((calcaneal or calcaneum or calcaneus or slide or heel or double) and osteotomy).mp. [mp =ti, ab, hw, tn, ot, dm, mf, dv, kw, fx, dq, nm, kf, ox, px, rx, ui, sy] 5 (fusion or arthrodesis).mp. [mp =ti, ab, hw, tn, ot, dm, mf, dv, kw, fx, dq, nm, kf, ox, px, rx, ui, sy] (601149) 6 Arthroereisis.mp. [mp =ti, ab, hw, tn, ot, dm, mf, dv, kw, fx, dq, nm, kf, ox, px, rx, ui, sy] (394) 7 (Grice Green or Grice-Green).mp. [mp =ti, ab, hw, tn, ot, dm, mf, dv, kw, fx, dq, nm, kf, ox, px, rx, ui, sy] (40) 8 2 or 3 or 4 or 5 or 6 (607577) 9 1 and 7 (1499)
